# Numerical investigation of the impact of neural stem cells on brain tumors

**DOI:** 10.1097/MD.0000000000051105

**Published:** 2026-10-16

**Authors:** Pius Erheyovwe Bubu, Val Hyginus Udoka Eze, Kelechi John Ukagwu, Vidya Sankarapandian, Saidi Odoma, Awafung Emmanuel Adie, Nansamba Fridah Mirembe

**Affiliations:** a Department of Biomedical Engineering, Kampala International University, Western Campus, Bushenyi Ishaka, Uganda; b Department of Electrical and Electronics Engineering, Mbarara University of Science and Technology, Kihumuro, Uganda; c Department of Electrical, Telecommunication, and Computer Engineering, Kampala International University, Western Campus, Bushenyi Ishaka, Uganda; d Department of Microbiology and Immunology, Kampala International University, Bushenyi Ishaka, Uganda; e Department of Pharmacology and Toxicology, Kampala International University, Western Campus, Bushenyi Ishaka, Uganda; f Department of Nursing Science, Kampala International University, Western Campus, Ishaka, Uganda.

**Keywords:** brain tumors, malignant brain tumor, neural stem cells (NSCs), numerical simulation, therapeutic potential, tumor dynamics

## Abstract

Malignant brain tumors, including glioblastoma and medulloblastoma, remain highly treatment-resistant. Neural stem cells (NSCs) are being investigated as tumor-tropic therapeutic vectors, but the temporal interaction between tumor and NSC populations requires quantitative characterization before model-based treatment hypotheses can be interpreted reliably. This study aimed to develop and numerically evaluate a reproducible 2-population model of tumor–NSC dynamics and to quantify the influence of NSC persistence and NSC-mediated tumor killing on simulated tumor burden. A normalized system of 2 coupled nonlinear ordinary differential equations was used: *dT*/*dt* = *rT*(1 − *T*) − β*T*N and *d*N/*dt* = αN(1 − N) − γ*T*N. The baseline simulation used *r* = 0.20000 day^−1^, α = 0.05000 day^−1^, β = 0.35000 day^−1^, γ = 0.30000 day^−1^, T_0_ = 0.50000, and N_0_ = 0.40000. Explicit Euler integration was performed with Δ*t* = 0.01 day over 20 days. A descriptive nonlinear regression fit, Fourier spectral analysis, 1-parameter sensitivity analyses, and joint α–β sensitivity analysis were applied to the simulated trajectory. In the baseline simulation, tumor density decreased from 0.5000 to a minimum of 0.4772 at day 2.59 (4.57% below the initial value) and subsequently increased to *T*_20_ = 0.8273, while NSC density declined from 0.4000 to N_20_ = 0.0247. The descriptive regression reproduced the simulated tumor trajectory with *R*^2^ = 0.9973 and root mean square error = 0.00590. Increasing α from 0.05 to 0.10 day^−1^ reduced *T*_20_ by 12.6% relative to baseline, and increasing β from 0.35 to 0.50 day^−1^ reduced *T*_20_ by 19.9%. Jointly setting α = 0.10 and β = 0.50 day^−1^ yielded *T*_20_ = 0.4427, 46.5% below the baseline terminal tumor density. The Fourier spectrum was dominated by the zero and low-frequency components, with no distinct oscillatory peak over the 20-day window. The model indicates that NSC persistence and NSC-mediated tumor killing materially influence terminal tumor burden, but the baseline parameterization produces only transient early suppression followed by tumor regrowth as the NSC population declines. The results are population-level, model-based estimates rather than tumor-type-specific clinical efficacy measures. Tumour-specific calibration, spatial migration, immune interactions, and experimental validation are required before translational conclusions can be drawn.

## Graphical abstract

**Figure:**
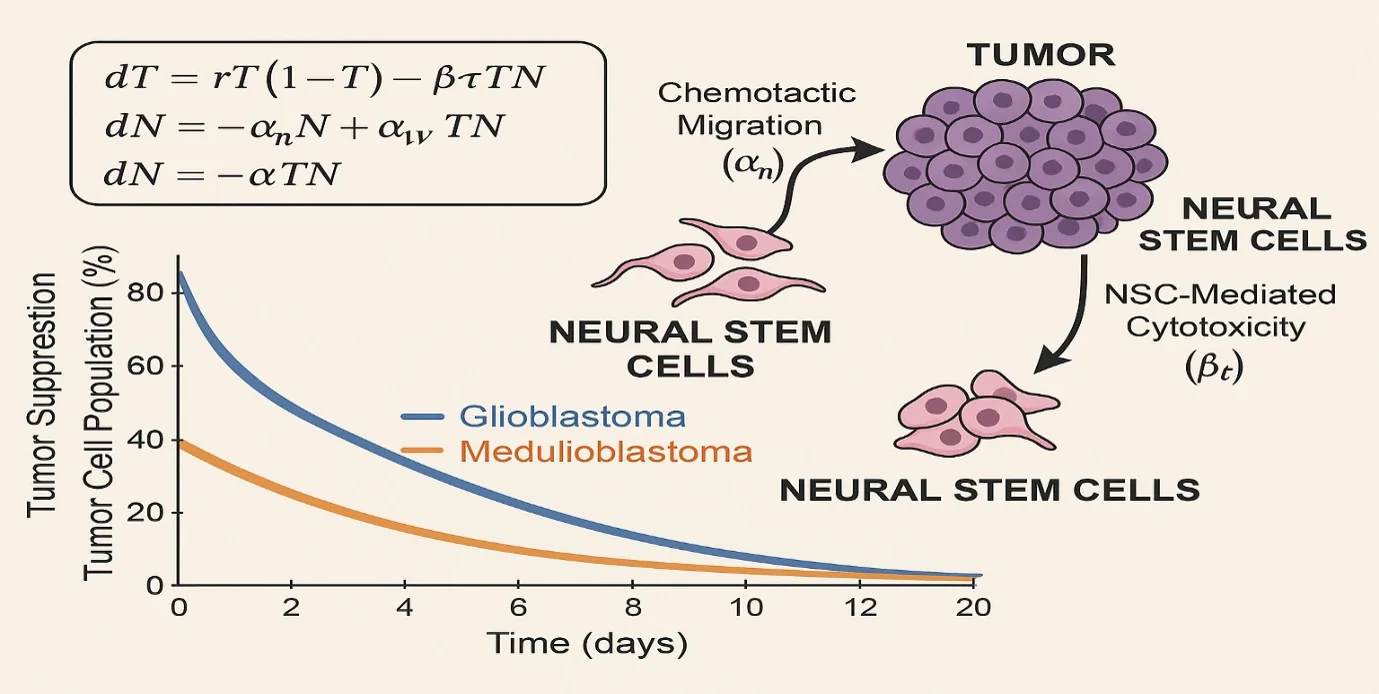

## 1. Introduction

Glioblastoma (GBM) remains one of the most lethal and treatment-refractory malignancies in neuro-oncology, characterized by its rapid proliferation, diffuse infiltration, and profound molecular and phenotypic heterogeneity. Classified by the World Health Organization as a grade IV astrocytoma, GBM is the most common primary malignant brain tumor in adults, with an annual incidence of approximately 3.2/100,000 individuals.^[1–7]^ Clinically, it is defined by hallmark features such as high mitotic activity, extensive angiogenesis, necrosis, and resistance to apoptosis-inducing treatments.^[8,9]^ The current standard of care, comprising maximal surgical resection followed by radiotherapy and concomitant temozolomide chemotherapy, yields a median overall survival of only 14 to 16 months, with 5-year survival rates below 5%.^[10–12]^ These grim outcomes are largely attributed to intrinsic and acquired treatment resistance, immune evasion, tumor recurrence, and the limited permeability of the blood–brain barrier (BBB), which collectively impede the efficacy of most systemic therapies.

Medulloblastoma, although predominantly a pediatric malignancy, shares several clinical and biological challenges with GBM, including invasive growth, dissemination through cerebrospinal fluid pathways, and treatment-induced neurotoxicity.^[2]^ Standard multimodal therapy involving surgical resection, craniospinal irradiation, and adjuvant chemotherapy has improved survival rates but is frequently associated with long-term neurocognitive impairment and reduced quality of life.^[3–5]^ Collectively, both glioblastoma and medulloblastoma exemplify the limitations of current therapeutic paradigms and highlight the urgent need for more targeted, effective, and biologically guided treatment modalities.

### 1.1. Neural stem cells as a targeted therapeutic platform

Neural stem cells (NSCs) have emerged as a novel and highly promising therapeutic platform for brain tumors, owing to their intrinsic tumor-tropic properties and amenability to genetic modification. NSCs are naturally guided to tumor foci by chemotactic cues such as stromal cell-derived factor-1, vascular endothelial growth factor, and hepatocyte growth factor secreted by the tumor microenvironment.^[13,14]^ This selective migratory behavior enables NSCs to home in on both primary and infiltrative tumor sites, features that conventional drug delivery systems often fail to achieve.

Furthermore, NSCs can be engineered to express or secrete a wide range of therapeutic agents, including prodrug-converting enzymes (e.g., cytosine deaminase), immune-modulatory cytokines (e.g., interleukin-12 (IL-12), interferon beta), and oncolytic viruses, thereby enhancing their tumoricidal capacity.^[15,16]^ Preclinical models have shown that NSC-based delivery systems can effectively cross the BBB, penetrate tumor-infiltrated brain regions, and induce significant reductions in tumor volume and prolongation of survival in both GBM and medulloblastoma models.^[17–21]^ These findings position NSCs not merely as passive delivery vehicles, but as active, adaptable agents capable of mounting a multifaceted and spatially targeted anti-tumor response.

### 1.2. Rationale for computational modeling of NSC–tumor interactions

Despite the compelling preclinical evidence supporting NSC-based therapies, their clinical translation necessitates a deeper mechanistic understanding of the spatiotemporal dynamics governing NSC–tumor interactions. These dynamics are driven by a complex interplay among tumor proliferation, NSC migration, cytotoxic activity, and environmental factors such as immune components and nutrient gradients. Empirical exploration of these interactions alone is limited by cost, ethical concerns, and experimental intractability, especially in the human brain. Thus, computational modeling offers a powerful complementary approach.

This study aims to develop a biologically informed and computationally robust mathematical model that captures the dynamic interplay between NSCs and brain tumor cells, specifically glioblastoma and medulloblastoma. The model represents tumor and NSC population dynamics using a system of 2 coupled nonlinear ordinary differential equations with logistic growth and bilinear interaction terms for NSC-mediated tumor-cell killing and tumor-induced NSC loss. Because the present model is nonspatial, chemotactic migration is not represented explicitly as a transport process or separate state variable. Key model parameters are examined through sensitivity analysis, while nonlinear regression and Fourier-based temporal analysis are used to characterize patterns in the simulated tumor response. By simulating various therapeutic conditions, this model serves as a predictive framework for evaluating NSC-based treatment strategies and their potential therapeutic efficacy. Ultimately, this modeling effort contributes to the rational design of future NSC-based therapies in neuro-oncology.

## 2. Literature review

NSCs have emerged as promising therapeutic agents for the treatment of brain tumors due to their inherent ability to target and eliminate tumorigenic cells. In neuro-oncology, NSCs are commonly employed as vectors for the targeted delivery of therapeutic agents, thereby overcoming the limitations of conventional treatments that fail to effectively cross the BBB.^[22]^ One of the pioneering studies in this field, reported in^,[14]^ demonstrated the intrinsic tumor-tropic properties of NSCs toward gliomas. The study further revealed that NSCs could be genetically engineered to express therapeutic genes, thereby enhancing the delivery of these genes to malignant cells.^[23]^

Subsequent research has focused on genetically modifying NSCs to improve their therapeutic efficacy. For example, the study in^[16]^ utilized NSCs engineered to express prodrug-activating enzymes capable of converting nontoxic prodrugs into cytotoxic agents within the tumor microenvironment. This strategy not only increased the selectivity of the treatment but also minimized collateral damage to healthy cells and tissues. Advancements have also been made in leveraging NSCs as delivery vehicles for oncolytic viruses, cytokines, and immune modulators.^[24]^ In^[20]^ researchers demonstrated the use of NSCs to transport oncolytic adenoviruses – viruses that selectively infect and lyse cancer cells – thereby enhancing the local concentration and efficacy of the therapeutic payload. These multifaceted strategies underscore the versatility of NSCs as a customizable therapeutic platform, capable of being tailored to target specific tumor characteristics and mechanisms of resistance.

### 2.1. Previous numerical and experimental studies on NSC–tumor interactions

Numerical and experimental approaches have significantly advanced our understanding of the complex interactions between NSCs and tumor cells, thereby informing the development of more effective therapeutic strategies. Among these approaches, mathematical modeling has emerged as a particularly valuable tool for describing NSC–tumor dynamics under varying biological and therapeutic conditions.^[25,26]^ A seminal study by researchers in^[19]^ was among the 1st to demonstrate the therapeutic potential of NSCs by using them to deliver the suicide gene thymidine kinase in combination with the prodrug ganciclovir, resulting in significant tumor regression in rodent models. This work provided a critical proof of concept for NSC-based therapies and highlighted the importance of integrating computational modeling with biological experimentation to optimize therapeutic strategies. Subsequent numerical studies have aimed to characterize the interactions between NSCs and tumor cells using mathematical models that incorporate key features of the underlying biological systems.^[26–32]^ For example, the work presented in^[27]^ developed a theoretical model describing NSC migration in response to chemotactic gradients secreted by glioma cells. Their system of coupled differential equations quantified the spatial and temporal distribution of migrating and proliferating NSCs, enabling an analysis of NSC–tumor interactions and laying the foundation for optimizing therapeutic delivery.^[25]^

More recent investigations have incorporated advanced modeling techniques such as agent-based modeling, nonlinear regression, and stochastic simulations to better capture the heterogeneity and randomness inherent in tumor–NSC interactions.^[28]^ These models have facilitated the evaluation of key therapeutic parameters, including NSC dosage, migration velocity, therapeutic gene expression, and their combined effects on tumor suppression. In addition, Fourier analysis has been employed to investigate the periodicity of NSC-mediated regulation of tumor proliferation, offering insights into the timing of tumor control and potential recurrence.^[29,33–38]^ The clinical implications of these findings are profound. The demonstrated ability of NSCs to modulate tumor growth kinetics underscores their potential as a platform for malignant brain tumor therapy. Simulation results suggest that enhancing NSC migratory capacity and therapeutic payload delivery can substantially improve treatment efficacy.^[21,24,39–45]^ Furthermore, mathematical sensitivity analyses indicate that individual patient characteristics, such as tumor microenvironment, molecular profile, and immune landscape, should inform the customization of NSC therapies. This aligns with the principles of personalized medicine, wherein therapeutic strategies are tailored to the genetic and physiological profile of each patient’s tumor.^[14,23,46–50]^ Finally, NSCs’ unique capacity to act as delivery vectors for diverse therapeutic agents, including oncolytic viruses, prodrug-activating enzymes, and immune modulators, greatly extends their clinical utility. This versatility allows NSCs to address the challenges posed by tumor heterogeneity and adaptive resistance, particularly in brain malignancies where conventional therapies often fail due to poor penetration of the BBB.

### 2.2. Gaps in the current knowledge

Nevertheless, several important issues still need to be addressed in the current literature concerning the potential of NSC-based therapies, which are crucial to be solved to derive the best out of NSCs for the treatment of brain tumors. Firstly, most of the prior research has been done on animals, and very few of them have been translated into clinical use. Although in vitro, ex vivo, and in vivo studies are promising at the initial stage, the differences between human brain tumors and patients’ responses to the treatments can be quite remarkable, and thus, large-scale clinical trials are needed to confirm the applicability and effectiveness of NSC-based therapies in the clinic.

Secondly, current models do not have characteristics like tumor heterogeneity, the immune system, and the BBB. The following are some of the factors that lead to the development of brain tumors and their consequences regarding therapy. Future models should try to include these factors to provide a real picture of the in vivo conditions. Further, the roles played by NSCs in the long run in the brain and the negative impact of NSCs have not been determined. Given the fact that NSCs are considered to be relatively safe, it is important to have a long-term assessment of NSCs’ behavior and their ability to transform into a malignant form or be rejected by the patient’s immune system in the long term.

Finally, more information concerning the methods of delivering NSCs and the appropriate dose schedules is required. Thus, the therapeutic potential of NSCs is proportional to the migration and infiltration of NSCs within the tumor mass. Hence, studies that focus on improving the migration, homing, and survival of NSCs in the tumor microenvironment are essential for attaining the best therapeutic effects. In summary, it is pertinent to state that there have been certain achievements in the development of NSC-based therapies for brain tumors; however, further research is crucial to fill the existing gaps and apply these promising approaches to clinical practice. Thus, the incorporation of experimental findings with enhanced computational models can help future research in identifying novel and personalized NSC-based therapies, which can help in providing better outcomes to those suffering from these fatal cancers.

## 3. Methodology

The interactions between tumor and NSC populations are represented using coupled logistic growth equations with bilinear interaction terms for NSC-mediated tumor-cell killing and tumor-induced NSC loss. This study, therefore, uses a 2-equation, 1st-order nonlinear ordinary differential equation (ODE) system to describe the temporal population dynamics of tumor cells and NSCs. The model is nonspatial; NSC chemotactic migration is not represented as a separate state variable or transport term.

### 3.1. Equations governing tumor and NSC population dynamics and NSC-mediated tumor-cell killing

The model used in this study comprises 2 coupled nonlinear ordinary differential equations for the tumor-cell population *T*(*t*) and NSC population N(*t*), given by Equations (1) and (2). Equations (3) and (4) in Section 3.3.1 are the explicit Euler discretizations of Equations (1) and (2), respectively, and therefore do not constitute a separate mathematical model.


$$\begin{array}{c} \frac{dT}{dt}=\alpha_{t}T\left(1-\frac{T}{K_{t}}\right)-\beta_{t}TN \end{array}$$ (1)

$$\begin{array}{c} \frac{dN}{dt}=\alpha_{n}N\left(1-\frac{N}{K_{n}}\right)-\beta_{n}TN \end{array}$$ (2)

where

*T*(*t*) is the normalized tumor density at time *t*; N(*t*) is the normalized NSC density at time *t*; αₜ = r, is the intrinsic tumor-proliferation rate; βₜ = β is the effective NSC-mediated tumor-killing coefficient; αₙ = α is the intrinsic NSC proliferation/persistence rate; βₙ = γ is the effective tumor-induced NSC-depletion coefficient. Because the state variables are normalized to their carrying capacities, Kₜ = Kₙ = 1, in the implemented dimensionless system.

Explanation of the functions of each component of Equations (1) and (2) is as follows:

1. Tumor cell growth and carrying capacity: The term αₜT(1 − T/Kₜ) represents logistic tumor growth. In the implemented normalized system Kₜ = 1, so the term is equivalently *rT*(1 − *T*), with r = αₜ.
2. NSC-mediated tumor-cell killing: The interaction term $-\beta_{t}TN$ models the cytotoxic effect of NSCs on tumor cells, where $-\beta_{t}$ is the rate at which NSCs induce tumor cell death. This term is crucial for capturing the therapeutic impact of NSCs on the tumor population.
3. NSC growth and carrying capacity: The term r = αₜ represents NSC proliferation/persistence. In the implemented normalized system Kₙ = 1, so the term is equivalently αN(1 − N), with α = αₙ.
4. Tumour-induced NSC death: The interaction term $-\beta_{n}TN$ accounts for the detrimental impact of the tumor on NSC viability, where $\beta_{n}$ is the rate at which the presence of tumor cells induces NSC death.

### 3.2. Parameters used in the model and their sources

To distinguish the exact values used in the reference simulation from literature-supported ranges, Table 1 reports the complete baseline parameter set, initial conditions, and numerical settings. The simulation was implemented in normalized form. The notation used in the optimization design corresponds to the governing equations as r = αₜ, α = αₙ, β = βₜ, and γ = βₙ. Literature ranges are retained for biological context, but they are not substitutes for the baseline values actually used.

**Table 1:** Exact baseline parameter values used in the tumor–NSC simulation, with literature-supported ranges.

Parameter	Description	Baseline value used	Range	Units/basis
αₜ (r)	Intrinsic tumor-proliferation rate	0.20000	0.05–0.20	day^−1^;^[30,31]^
αₙ (α)	Intrinsic NSC proliferation/persistence rate	0.05000	0.02–0.10	day^−1^;^[14,33,34]^
βₜ (β)	Effective NSC-mediated tumor-killing coefficient	0.35000	0.08–0.50	day^−1^;^[19,32]^
βₙ (γ)	Effective tumor-induced NSC-depletion coefficient	0.30000	0.05–0.35	day^−1^;^[16,19]^
*T*_0_	Initial normalized tumor density	0.50000	0.10–0.90	Dimensionless; assumed
N_0_	Initial normalized NSC density	0.40000	0.10–0.90	Dimensionless; assumed
Kₜ, Kₙ	Normalized carrying capacities	1.00000 each	Fixed	Dimensionless; normalization
Δ*t*	Euler integration time step	0.01000	Fixed	day
*t*_max_	Simulation horizon	20.00000	Fixed	days
n	Euler update steps	2000	Fixed	steps

NSC = neural stem cell.

Note: Baseline values are the exact values used for the reference simulation. The ranges shown are literature-supported or assumed design ranges. Because *T* and N are normalized, the carrying capacities are unity in the implemented equations. The artificial intelligence-optimized parameter set is not used as the baseline and is reported separately in the optimization analysis.

### 3.3. Simulation setup

The baseline simulation was initialized using the normalized initial conditions reported in Table 1:

1. Initial normalized tumor density (*T*_0_): 0.50000 (dimensionless). Under the reference scaling previously used for presentation (tumor capacity 2000 cells), this corresponds to 1000 tumor cells.
2. Initial normalized NSC density (N_0_): 0.40000 (dimensionless). Under the reference scaling previously used for presentation (NSC capacity 500 cells), this corresponds to 200 NSCs.

The model was simulated over a 20-day horizon using Δ*t* = 0.01 day, corresponding to 2000 explicit Euler update steps after initialization. These numerical settings, together with the full baseline parameter set, are reported explicitly in Table 1 for reproducibility.

#### 3.3.1. Implementation

The numerical implementation uses the explicit Euler method to advance the same 2-equation system defined by Equations (1) and (2). In the normalized implementation, Kₜ = Kₙ = 1 and the parameter mapping is αₜ = r, αₙ = α, βₜ = β, and βₙ = γ. Equation (3) discretizes the tumor equation and Equation (4) discretizes the corrected NSC equation; therefore, Equations (3) and (4) are update formulas for the same continuous-time model rather than a 2nd model.


$$\begin{array}{c} T_{t+1}=T_{t}+\left(\alpha_{t}T_{t}\left(1-\frac{T_{t}}{K_{t}}\right)-\beta_{t}T_{t}N_{t}\right)\times dt \end{array}$$ (3)

$$\begin{array}{c} N_{t+1}=N_{t}+\left(\alpha_{n}N_{t}\left(1-\frac{N_{t}}{K_{n}}\right)-\beta_{n}T_{t}N_{t}\right)\times dt \end{array}$$ (4)

For clarity, the normalized equations implemented in the baseline simulation are *dT*/*dt* = *rT*(1 − *T*) − β*T*N and *d*N/*dt* = αN(1 − N) − γ*T*N. With *r* = 0.20000, α = 0.05000, β = 0.35000, γ = 0.30000, *T*_0_ = 0.50000, and N_0_ = 0.40000, the reference trajectory is generated by iterating the corresponding Euler updates with Δ*t* = 0.01 day.

### 3.4. Model assumptions and parameter calibration

The mathematical model describing the dynamics of tumor cells and neural stem cells is governed by 2 coupled, nonlinear ordinary differential equations. The interaction terms between tumor cells and NSCs in Equations (1) and (2) represent biologically relevant processes of NSC-mediated tumor-cell killing and tumor-induced NSC loss. The formulation is built upon several key assumptions and methodologies for parameter selection. The numerical update scheme is specified by Equations (3) and (4), while Appendix A, Supplemental Digital Content 1 presents the subsequent regression, Fourier analysis, and plotting workflow applied to the generated time-series data.

#### 3.4.1. Assumptions underlying the model

1. Logistic growth dynamics: Both state variables are normalized to their respective carrying capacities; hence, K ₜ = K ₙ = 1 in the implemented system. The logistic terms therefore reduce to *rT*(1 − *T*) for tumor growth and αN(1 − N) for NSC proliferation/persistence.
2. Bilinear interaction terms: The terms (−β_*t*_*T*N and −β_n_*T*N) capture pairwise interactions between tumor cells and NSCs, assuming that:

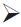


The probability of interaction is proportional to the product of the 2 populations.

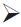


The effects are symmetric in mathematical form but asymmetric in biological effect, with NSCs killing tumor cells and tumor cells inducing NSC death.
3. Homogeneous mixing assumption: The model assumes a well-mixed tumor microenvironment, such that all NSCs and tumor cells have equal probabilities of interacting.
4. Constant parameter values: Parameters are time-invariant and do not adapt dynamically to therapy, immune responses, or microenvironmental changes. This simplifies calibration but may limit long-term prediction accuracy.

#### 3.4.2. Parameter selection and calibration

The exact baseline values used in the reference simulation are reported in Table 1. Rate parameters were selected within literature-supported ranges, while the normalized initial conditions were specified within the assumed design range. Reporting both the exact baseline and the contextual ranges separates the reproducible simulation inputs from the literature used to motivate them.

#### 3.4.3. Calibration methodology

- Literature mapping: Literature-supported ranges were used to define biologically plausible intervals for *r*, α, β, and γ, as summarized in Table 1.
- Baseline specification: The reference simulation used *r* = 0.20000, α = 0.05000, β = 0.35000, γ = 0.30000, *T*_0_ = 0.50000, and N_0_ = 0.40000. These values are now reported explicitly to permit direct numerical reproduction.
- Sensitivity analysis: Conducted to assess the influence of each parameter on tumor suppression outcomes and to prioritize biologically significant interactions.

## 4. Results

The simulation results revealed the temporal interaction between NSC and tumor-cell populations and enabled quantitative analysis of the therapeutic effect represented by the model. The mathematical model, based on differential equations describing tumor and NSC population growth together with reciprocal interaction terms, produces the time course of tumor and NSC numbers under different scenarios.

### 4.1. Tumor and NSC dynamics over time

The simulated trajectories of NSCs and tumor cells provide insight into the temporal population dynamics represented by the 2-equation model. Tumor and NSC populations evolve according to their respective logistic-growth terms and the bilinear interaction terms in Equations (1) and (2). In this nonspatial formulation, N(*t*) denotes the total NSC population available to interact with tumor cells; explicit chemotactic migration, spatial gradients, and transport across the BBB are not modeled. Accordingly, the simulated trajectories should be interpreted as population-level temporal dynamics rather than direct simulations of spatial NSC homing.

Figure 1 shows the reproducible baseline trajectory generated from the parameter values in Table 1. Tumor density decreases from *T*_0_ = 0.5000 to a minimum of 0.4772 at day 2.59, corresponding to a 4.57% transient reduction, before regrowing to *T*_20_ = 0.8273. Over the same interval, NSC density decreases from N_0_ = 0.4000 to N_20_ = 0.0247. Thus, the baseline parameterization represents transient early tumor suppression followed by regrowth as the NSC population is depleted.

**Figure 1.**
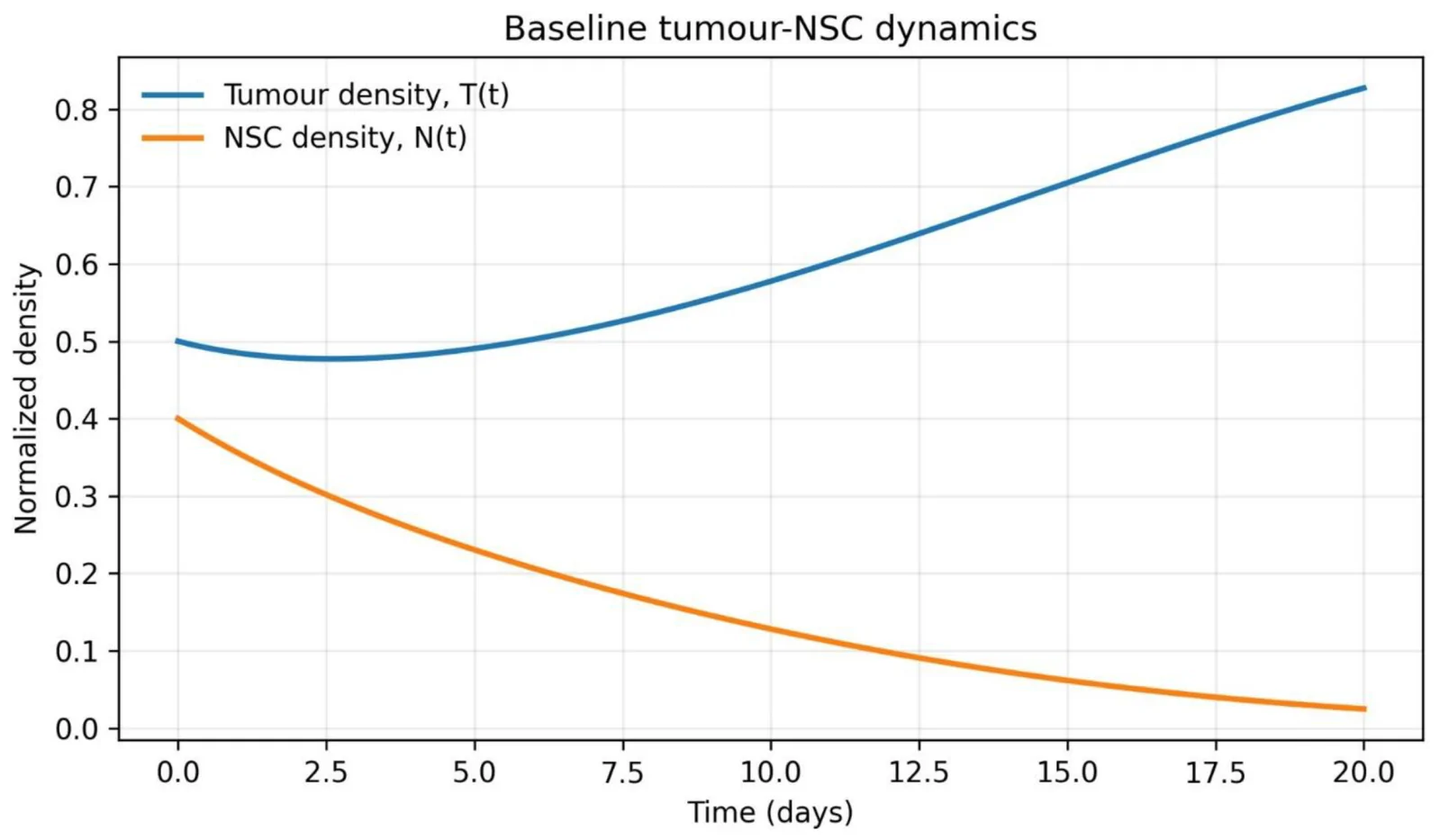
Baseline normalized tumor and NSC densities over 20 days. Tumour density reaches a minimum of 0.4772 at day 2.59 before increasing to 0.8273 at day 20; NSC density declines to 0.0247. NSC = neural stem cell.

Because the implemented model contains a single generic tumor state variable and no tumor-type-specific parameter sets, it does not support separate quantitative efficacy estimates for glioblastoma and medulloblastoma. Tumor-type comparisons therefore require independent calibration of *r*, α, β, γ, and the initial conditions using disease-specific experimental data.

#### 4.1.1. Nonlinear regression fit of the baseline tumor trajectory

Figure 2 compares the simulated baseline tumor trajectory with the descriptive nonlinear regression function used in Appendix A, Supplemental Digital Content 1. The fit yielded *R*^2^ = 0.9973 and root mean square error = 0.00590 in normalized-density units. This high within-series agreement indicates that the regression function closely summarizes the deterministic simulated trajectory; it should not be interpreted as independent biological validation because the fitted values are derived from the same simulated time series.

**Figure 2.**
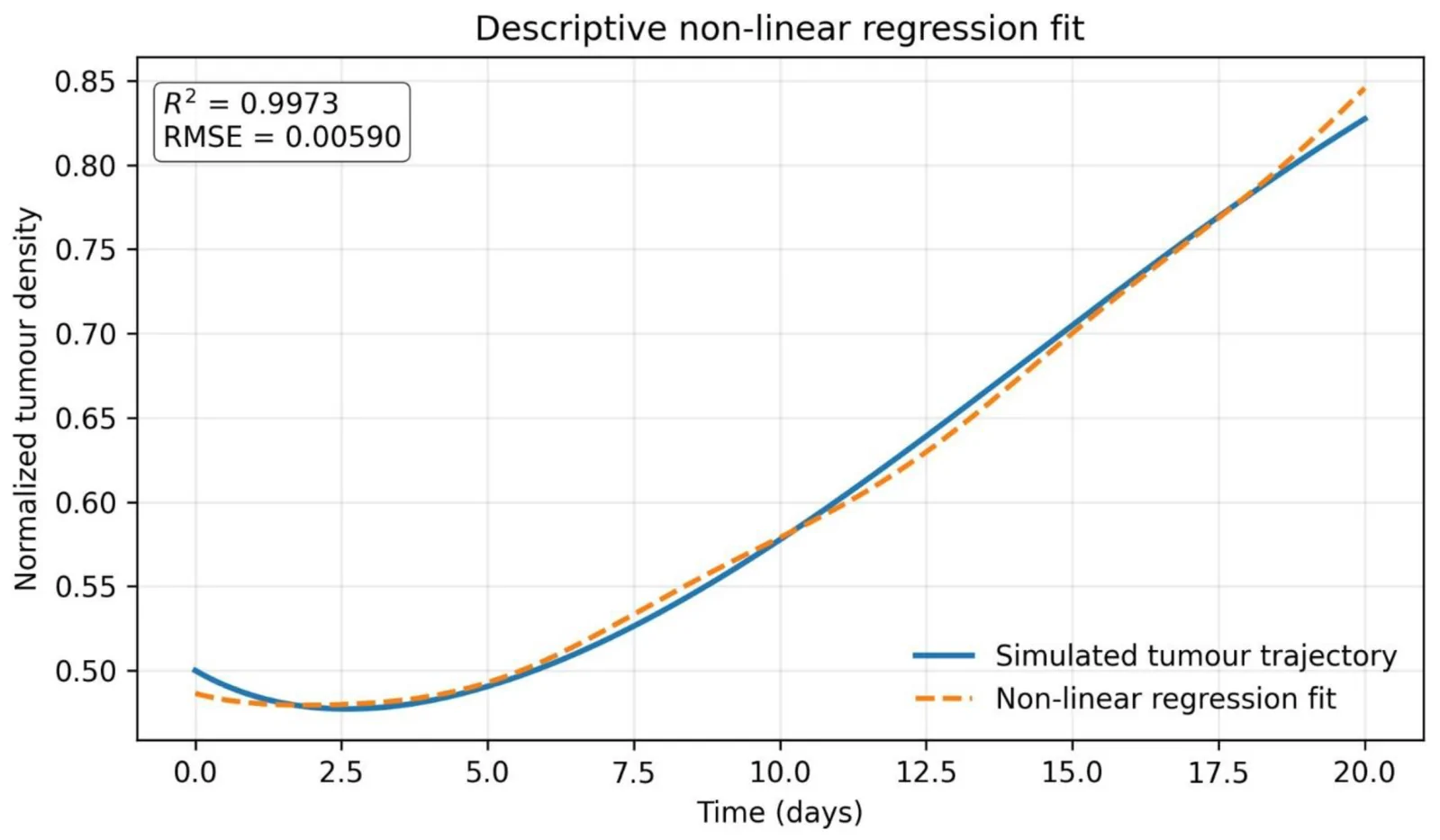
Descriptive nonlinear regression fit to the simulated baseline tumor trajectory (*R*^2^ = 0.9973; RMSE = 0.00590). The fit summarizes the same deterministic simulation and is not an independent validation dataset. RMSE = root mean square error.

#### 4.1.2. Fourier analysis of tumor-cell dynamics

To elucidate the temporal characteristics of tumor suppression mediated by NSC therapy, a Fourier transform was applied to the tumor cell population time series, enabling decomposition of the complex temporal dynamics into constituent frequency components.

The 1-sided Fourier amplitude spectrum in Figure 3 is dominated by the direct-current (zero-frequency) and lowest-frequency components, with amplitude decreasing as frequency increases. Frequency is reported in day^−1^ because the simulated time axis is measured in days. No distinct secondary spectral peak indicative of sustained periodic oscillation is present within the 20-day observation window. The spectrum should therefore be interpreted as evidence that the trajectory is governed primarily by slow trend components rather than a resolved periodic cycle.

**Figure 3.**
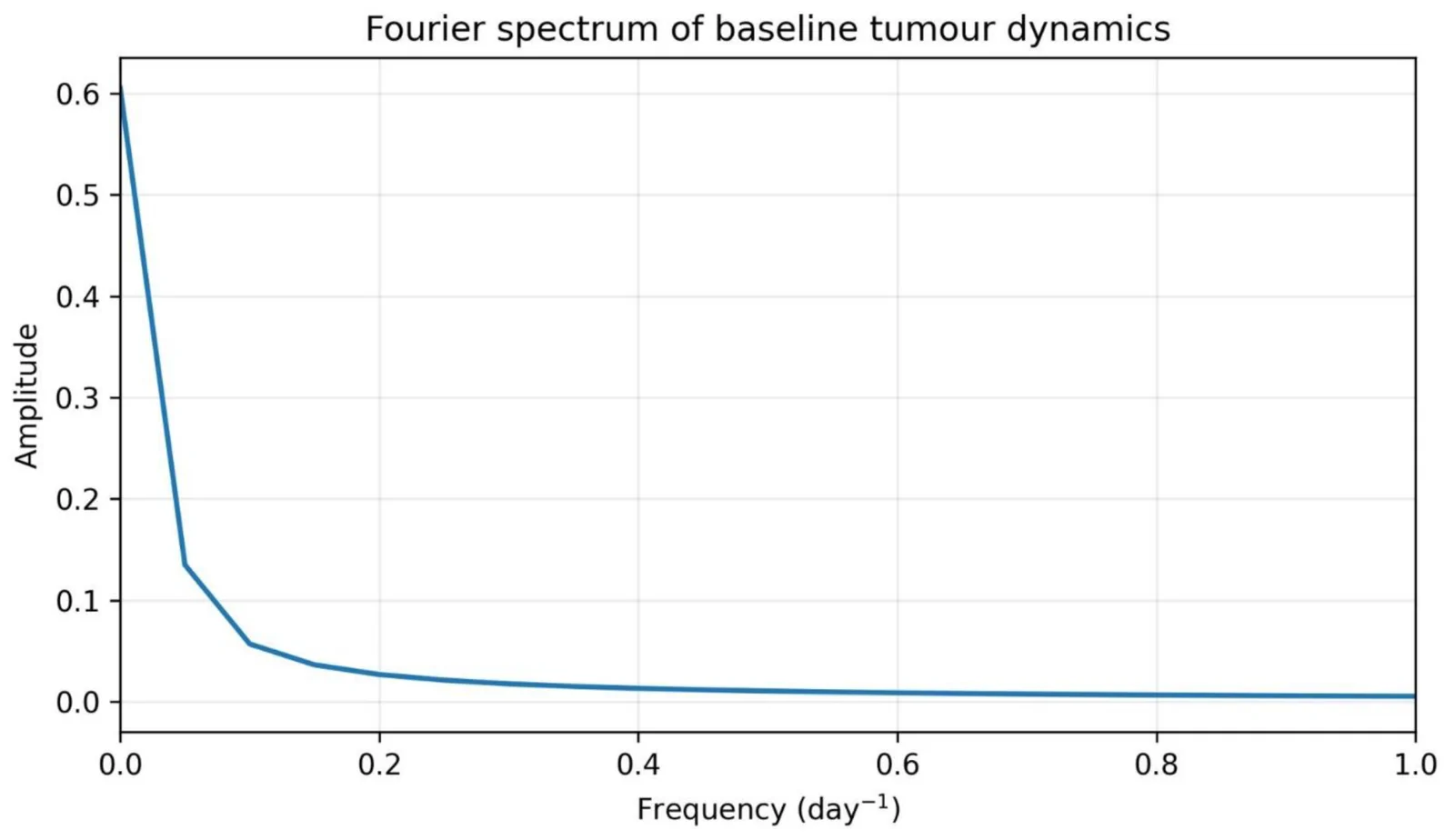
One-sided Fourier amplitude spectrum of the baseline tumor trajectory. The zero-frequency and low-frequency components dominate; frequency is expressed in day^−1^ because the time variable is measured in days.

Fourier analysis here is descriptive of the simulated time series and does not establish an intrinsic biological rhythm. Testing periodic NSC dosing or tumor-cell-cycle forcing would require an explicitly time-dependent input and a dedicated simulation design rather than inference from the baseline spectrum alone.

#### 4.1.3. Analysis of the impact of varying key parameters

The impact of key model parameters, particularly the NSC intrinsic growth rate (αₙ) and tumor cell kill rate (βₜ), was systematically examined through sensitivity analysis. These analyses help identify the parameters that most strongly influence tumor suppression within the 2-equation model.

##### 4.1.3.1. NSC growth rate (αₙ)

The intrinsic NSC growth rate, denoted by αₙ, controls the logistic proliferation term αₙN(1 − N/Kₙ) in Equation (2). Increasing αₙ increases the rate at which the NSC population can replenish, subject to the carrying capacity Kₙ, and therefore changes the number of NSCs available to interact with tumor cells through the modeled cytotoxic term.

Figure 4 presents a 1-parameter sensitivity analysis in which α was varied over 0.02, 0.05, 0.08, and 0.10 day^−1^, spanning the literature-supported interval in Table 1 while all other parameters were fixed at baseline values. At the baseline α = 0.05 day^−1^, T_20_ = 0.8273; increasing α to 0.10 day^−1^ reduced *T*_20_ to 0.7227, a 12.6% reduction relative to the baseline terminal value. The result indicates that greater NSC proliferation/persistence lowers terminal tumor burden in this model, although *T*_20_ remains above the initial tumor density at α = 0.10 day^−1^.

**Figure 4.**
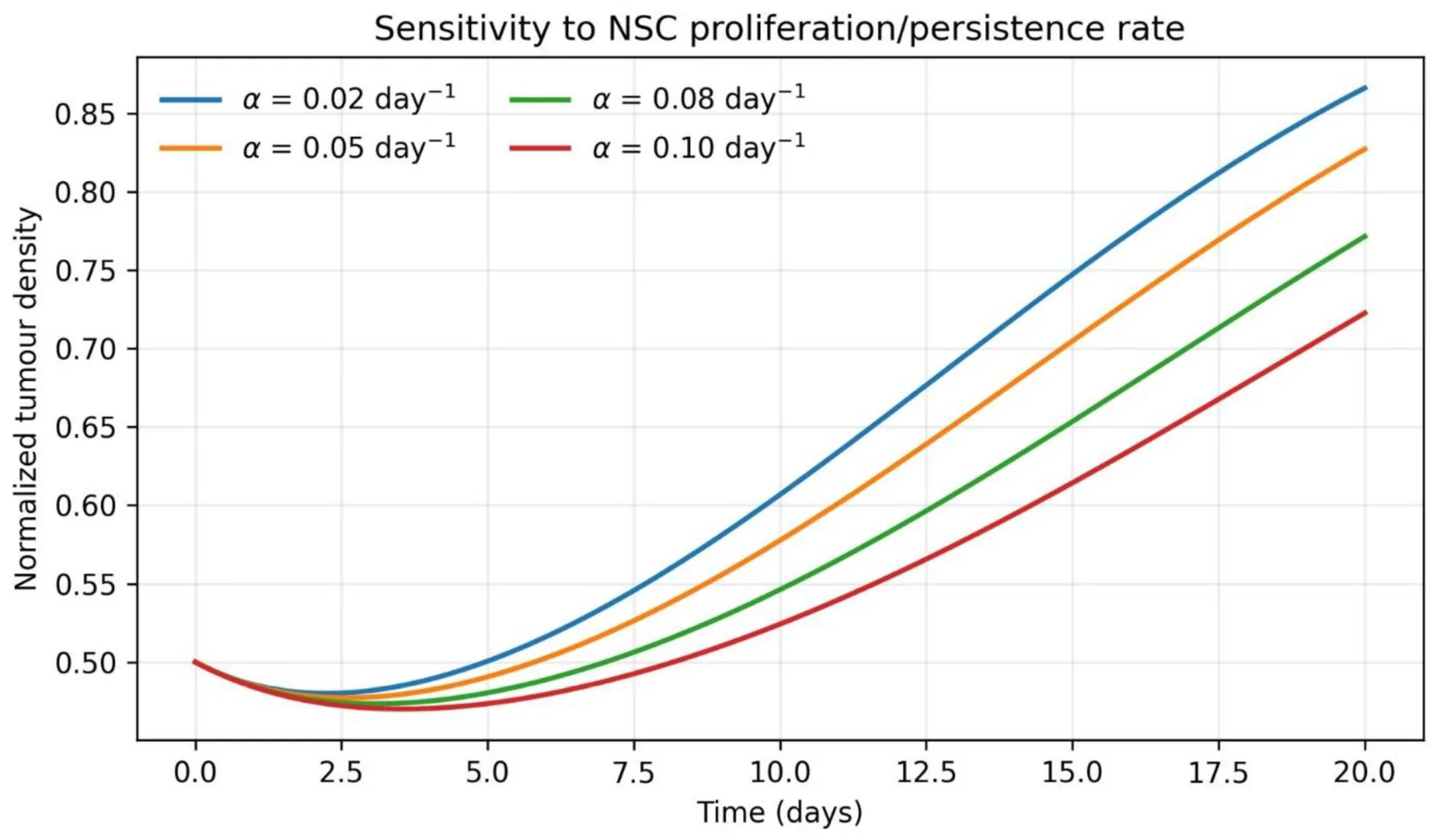
Sensitivity to the NSC proliferation/persistence rate α. Increasing α from the baseline 0.05 to 0.10 day^−1^ lowers *T*_20_ from 0.8273 to 0.7227 (12.6% relative reduction). NSC = neural stem cell.

#### 4.1.4. NSC-mediated tumor-killing coefficient (β)

The coefficient β controls the bilinear tumor-loss term βTN and therefore represents the effective strength of NSC-mediated tumor-cell killing per tumor–NSC interaction in the normalized model.

Figure 5 varies β over 0.08, 0.20, 0.35, and 0.50 day^−1^ while holding the remaining parameters at baseline values. Increasing β from the baseline value of 0.35 to 0.50 day^−1^ reduced *T*_20_ from 0.8273 to 0.6625, corresponding to a 19.9% relative reduction in terminal tumor density. The parameter therefore has a substantial influence on modeled tumor burden, but the result is a sensitivity estimate rather than direct evidence that an equivalent biological increase can be achieved safely in vivo.

**Figure 5.**
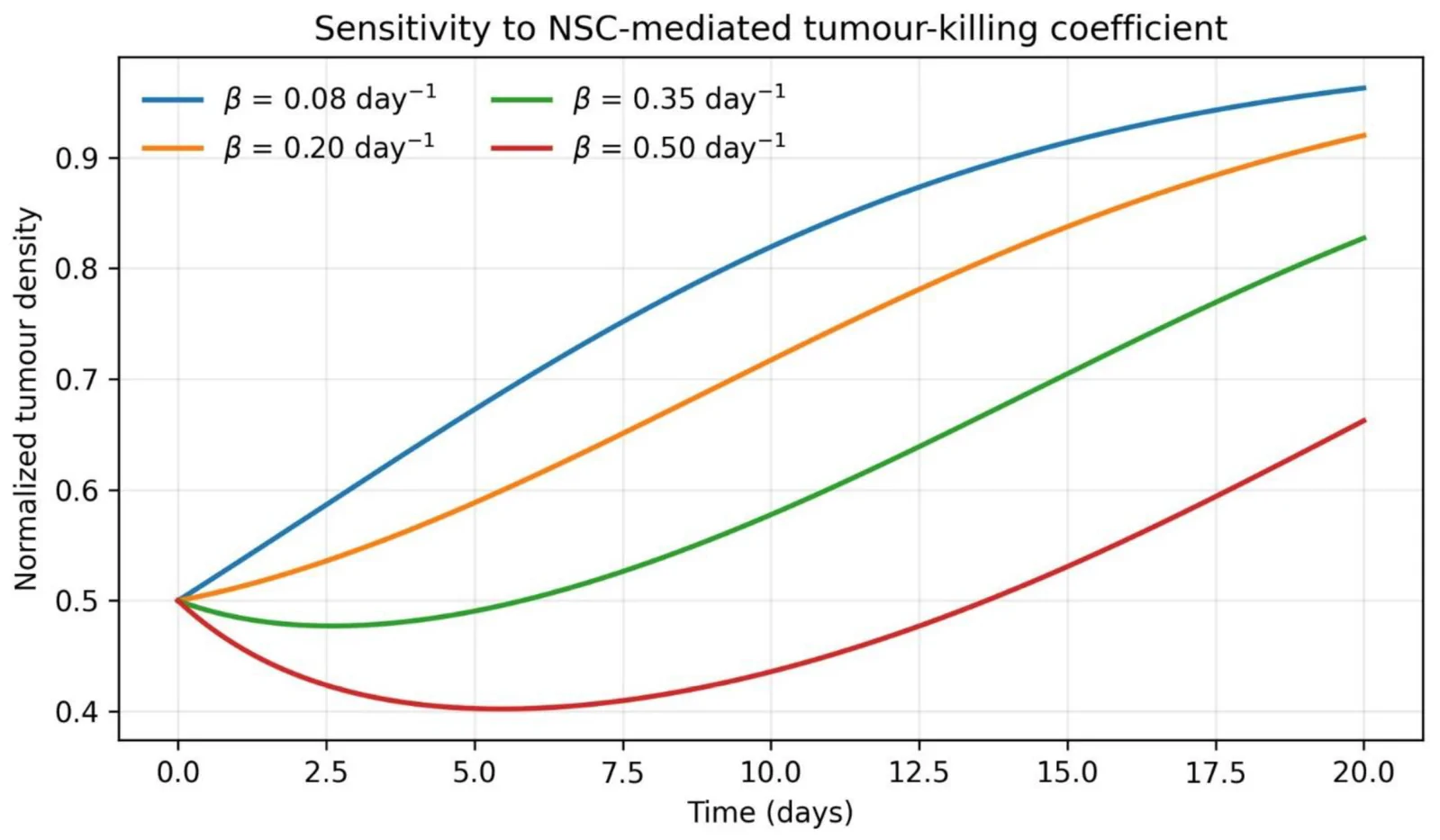
Sensitivity to the NSC-mediated tumor-killing coefficient β. Increasing β from the baseline 0.35 to 0.50 day^−1^ lowers *T*_20_ from 0.8273 to 0.6625 (19.9% relative reduction). NSC = neural stem cell.

The response to β is nonlinear because tumor and NSC densities co-evolve, and the interaction term depends on their product. Interpretation should therefore remain within the mathematical model: spatial transport, immune toxicity, pharmacokinetics, and dose-limiting biological effects are not represented explicitly.

#### 4.1.5. Combination of parameters

Figure 6 maps terminal tumor density *T*_20_ across the joint literature-supported α–β parameter domain while *r*, γ, *T*_0_, and N_0_ remain fixed at their baseline values. The baseline point (α = 0.05, β = 0.35 day^−1^) gives *T*_20_ = 0.8273. At α = 0.10 and β = 0.50 day^−1^, *T*_20_ decreases to 0.4427, which is 46.5% below the baseline terminal value and 11.5% below the initial tumor density *T*_0_ = 0.5000.

**Figure 6.**
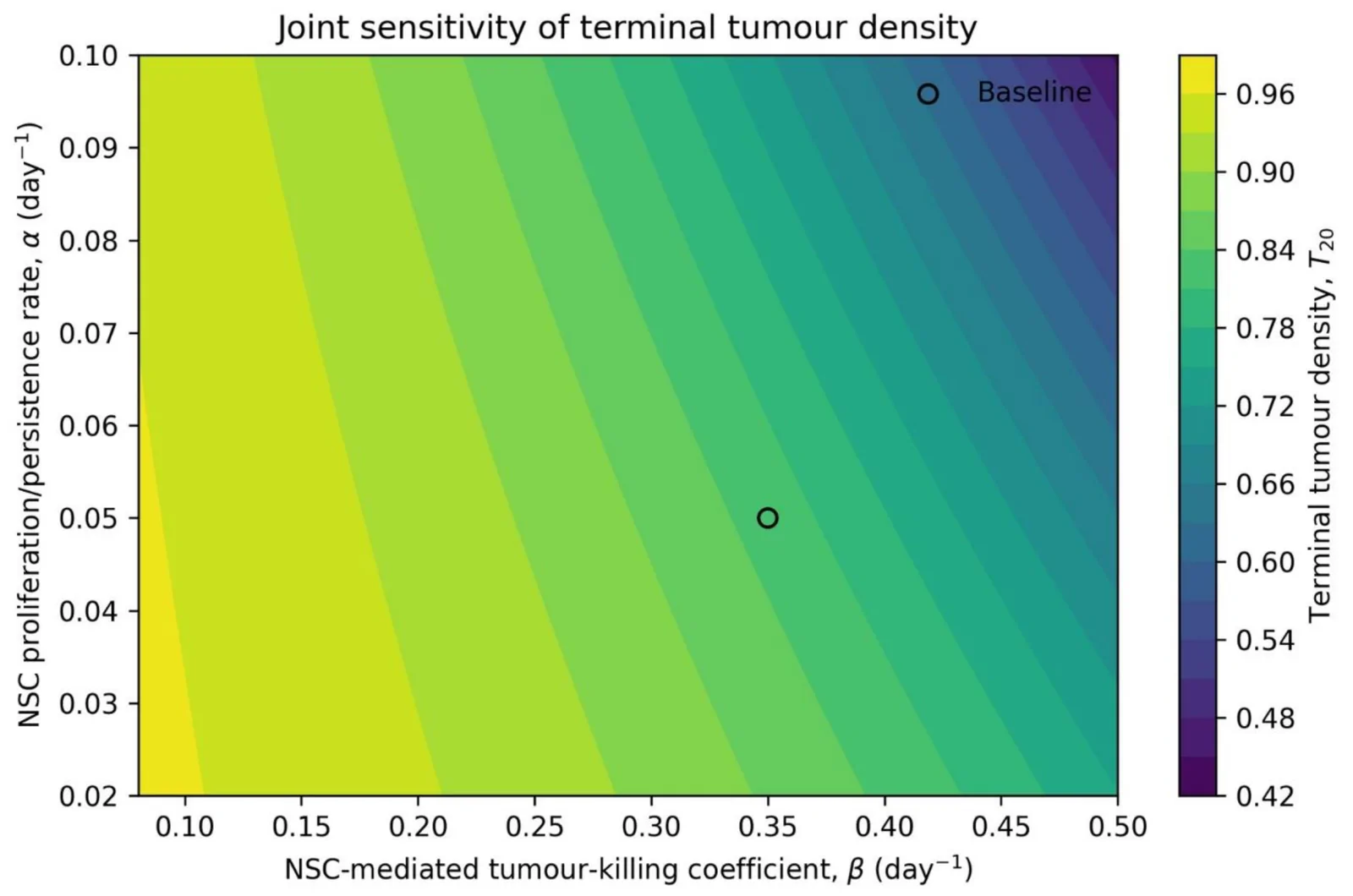
Joint sensitivity of terminal tumor density *T*_20_ to α and β. With other baseline parameters fixed, α = 0.10 and β = 0.50 day^−1^ give *T*_20_ = 0.4427, 46.5% below the baseline terminal density. The map represents model-based sensitivity, not clinical efficacy. NSC = neural stem cell.

For comparison, the separate changes α: 0.05 → 0.10 and β: 0.35 → 0.50 reduce *T*_20_ by 12.6% and 19.9%, respectively. Their combined change produces a larger 46.5% reduction, demonstrating a nonadditive joint effect within this nonlinear ODE system. This mathematical interaction should not be equated with clinical synergy without experimental validation.

### 4.2. Discussion

This study presents a computational investigation of tumor–NSC interactions using 2 coupled nonlinear ODEs with logistic population growth and reciprocal bilinear interaction terms. Under the reproducible baseline parameterization, tumor density falls only modestly to 0.4772 before regrowing to 0.8273 by day 20 as NSC density declines to 0.0247. This behavior is consistent with a transient cytotoxic effect that is not sustained when the therapeutic cell population is progressively depleted. The model does not contain separate glioblastoma and medulloblastoma parameterizations; consequently, tumor-type-specific percentage reductions cannot be inferred from the present simulations.

Sensitivity analysis shows that increasing α from 0.05 to 0.10 day^−1^ lowers terminal tumor density by 12.6% relative to baseline, whereas increasing β from 0.35 to 0.50 day^−1^ lowers it by 19.9%. Raising both parameters to those upper values gives *T*_20_ = 0.4427, a 46.5% reduction relative to the baseline terminal value. These are deterministic model comparisons; means, standard deviations, and *P* values are not reported because the present simulation does not define replicate stochastic realizations or an experimental sampling distribution.

The Fourier spectrum is dominated by the direct-current (zero-frequency) and low-frequency components and contains no distinct oscillatory peak over the 20-day window. This supports a description of the baseline trajectory as slow, trend-dominated dynamics rather than a resolved periodic process. The spectral result does not by itself establish an optimal dosing schedule. More realistic inference would require models that include time-dependent dosing, spatial migration, immune interactions, tumor heterogeneity, BBB effects, and calibration against longitudinal experimental data.

Furthermore, addressing the multifaceted challenges imposed by the tumor microenvironment is important for improving the translational efficacy of NSC-based therapies against malignant brain tumors such as glioblastoma and medulloblastoma.^[25,50–55]^ Potential strategies discussed in the broader literature include genetic modification of NSCs to alter immune signaling, biomaterial encapsulation to improve NSC survival, preconditioning approaches that enhance stress tolerance, and combination with systemic immunotherapies. These mechanisms are not represented in the present 2-equation model and therefore should be regarded as directions for future experimental and computational investigation rather than outputs of the simulations reported here. Recent chimeric antigen receptor T cell studies in hematologic malignancies illustrate engineering strategies that may inform future cell-based combination and safety-design work, including target-gene editing to reduce fratricide and exhaustion, nanobody-based chimeric antigen receptor construction, and a truncated epidermal growth factor receptor safety-switch approach.^[56–58]^ These studies did not investigate NSC therapy or brain tumors and are cited here as methodological precedents rather than as direct evidence for the present tumor–NSC model.

While the presented computational model offers valuable insights into tumor–NSC interactions, several limitations must be addressed to enhance its clinical applicability and predictive accuracy. Notably, the model omits immune cell interactions within the tumor microenvironment, despite evidence that immune components such as cytokines (e.g., tumor necrosis factor alpha, IL-6), regulatory T cells, and myeloid-derived suppressor cells significantly modulate NSC viability and function.^[40,41]^ Incorporating immunological feedback, including NSC-mediated immune modulation via engineered anti-inflammatory cytokines or checkpoint inhibitors (e.g., IL-10, programmed death-ligand 1 blockade), could improve therapeutic realism.^[42,43]^ The model also assumes fixed NSC dosing and administration schedules, limiting exploration of adaptive treatment strategies responsive to tumor progression or host immune status. Integrating pharmacokinetics-driven or agent-based dosing modules would better reflect clinical variability.^[44]^ Furthermore, the model simplifies NSC populations as homogeneous entities, overlooking inter-subpopulation heterogeneity in migratory behavior, immunogenicity, and therapeutic gene expression, as revealed by single-cell transcriptomic analyses.^[45–48]^ Future iterations should incorporate single-cell RNA sequencing-informed parameters to capture this functional diversity. Another critical gap is the exclusion of spatiotemporal NSC dynamics posttransplantation, which are essential for validating predicted tumor-homing and payload delivery behaviors. Embedding imaging-derived migration maps and real-time tracking data, such as from intravital microscopy and magnetic resonance imaging-compatible reporter systems, would enhance spatiotemporal resolution.^[49,50]^ In addition, the model does not account for dynamic BBB permeability, a key determinant of NSC infiltration. Incorporating BBB transport modules that reflect delivery routes and inflammatory or pathological cues would improve simulation fidelity.^[51]^ Finally, tumor clonal evolution under therapeutic pressure is not modeled, despite its role in resistance development. Incorporating stochastic mutation landscapes and evolutionary dynamics, guided by next-generation sequencing data, would enable prediction of escape trajectories and inform multiplexed NSC payload designs.^[52,53]^ Although prior models have explored NSC behavior (e.g., Gomez et al^[25]^ used agent-based simulations for tumor-tropic migration and Youssef et al^[54]^ applied differential equation-based frameworks for NSC-mediated oncolytic virus delivery), these lack integration of immune dynamics, BBB transport, and cellular heterogeneity. The present 2-equation model does not incorporate tumor subpopulation heterogeneity, immune dynamics, BBB transport, or explicit spatial migration; these are priorities for future refinement using hybrid frameworks with real-time spatial tracking and immune feedback loops. In particular, models such as that of de Montigny et al,^[55]^ which integrate spatial migration with immune checkpoint signaling, represent promising directions. Thus, future refinements should prioritize immune–NSC interactions, adaptive NSC dosing, heterogeneity-informed modeling, BBB dynamics, spatiotemporal tracking, and clonal resistance evolution to improve translational relevance.

## 5. Conclusion

This study develops a reproducible, normalized 2-population model for examining tumor–NSC dynamics. With the baseline parameters reported in Table 1, the model produces a transient 4.57% early reduction in tumor density followed by regrowth to *T*_20_ = 0.8273 as the NSC population falls to N_20_ = 0.0247. Increasing NSC proliferation/persistence (α) and the NSC-mediated tumor-killing coefficient (β) reduces terminal tumor burden, and their joint increase to α = 0.10 and β = 0.50 day^−1^ yields *T*_20_ = 0.4427, 46.5% below the baseline terminal value. Fourier analysis indicates low-frequency, nonperiodic dynamics over the simulated interval. These results identify α and β as influential model parameters but do not establish tumor-type-specific clinical efficacy because the current system contains 1 generic tumor state and omits spatial migration, immune feedback, pharmacokinetics, tumor heterogeneity, and patient-specific calibration. Experimental validation and tumor-specific parameter estimation are therefore required before the framework can support translational or personalized treatment predictions.

### 5.1. Limitations of the current study and suggestions for future research

However, several limitations of the current study should be noted despite the evidence supporting NSC-based therapies’ effectiveness. First of all, it is necessary to note that while being numerically very precise, the model does not capture some aspects of the tumor microenvironment, for instance, immune responses, tumor heterogeneity, and the effect of the BBB. It is suggested that future models should include these elements to mimic in vivo conditions and provide a better prognosis of the therapeutic interventions.

Also, the study mainly concentrates on the early-stage interactions of NSCs and tumors. The long-term consequences of the treatment remain uncertain and may be detrimental to the patient; for example, NSCs may be rejected by the immune system or undergo malignant transformation. In silico and in vivo longitudinal investigations are crucial for the proper assessment of NSC-based treatments’ safety and efficacy in the long term.

However, applying these discoveries from preclinical models to clinical practice requires more clinical trials. The effects of genetic, environmental, and lifestyle differences on the patient’s response to the treatment must be managed systematically to guarantee that the observed therapeutic effects in simulations and preclinical studies are reproducible. Further studies should also examine the synergistic application of NSCs with other forms of treatment like chemo- and radiotherapy, immunotherapy, and others. The enhancing effects of such combinations could also improve therapeutic efficacy and offer a comprehensive strategy to address brain tumors.

## Author contributions

**Conceptualization:** Pius Erheyovwe Bubu, Val Hyginus Udoka Eze.

**Methodology:** Val Hyginus Udoka Eze, Nansamba Fridah Mirembe.

**Resources:** Awafung Emmanuel Adie.

**Validation:** Saidi Odoma, Nansamba Fridah Mirembe.

**Writing – original draft:** Pius Erheyovwe Bubu, Val Hyginus Udoka Eze, Kelechi John Ukagwu, Vidya Sankarapandian, Saidi Odoma, Awafung Emmanuel Adie, Nansamba Fridah Mirembe.

**Writing – review & editing:** Pius Erheyovwe Bubu, Val Hyginus Udoka Eze, Kelechi John Ukagwu, Vidya Sankarapandian, Awafung Emmanuel Adie, Nansamba Fridah Mirembe.


