# Supplementary material for: Numerical investigation of the impact of neural stem cells on brain tumors
Source: Medicine (Baltimore). 2026 Oct 16;105(42):e51105. doi: 10.1097/MD.0000000000051105 (PMC13681089; doi:10.1097/MD.0000000000051105)
Supplement: Supplementary file 1 [file medi-105-e51105-s001.docx]

**Appendix A: Regression Functions Python codes, Fourier analysis, and plotting workflow**

import numpy as np

import pandas as pd

import matplotlib.pyplot as plt

from scipy.optimize import curve_fit

from scipy.fft import fft, fftfreq

from docx import Document

from docx.shared import Inches

\# The CSV below contains the tumour and NSC time series generated by iterating Equations (3) and (4).

\# LOAD SIMULATED TIME-SERIES FOR POST-PROCESSING

data_path = 'C:/Users/DrPius/Documents/Neural Stem Cell_vs_Brain tumours/tumour_nsc_dynamics_20days.csv'

df = pd.read_csv(data_path)

\# Extract data

time = df['Time (days)'].values

tumour_cells = df['Tumour Cells'].values

nscs = df['NSCs'].values

\# NON-LINEAR REGRESSION FITTING

def model_func(X, a, b, c, d, e, f):

t, n = X

return a * t + b * n + c * np.sin(d * t) + e * np.cos(f * n)

X_data = np.vstack((time, nscs))

y_data = tumour_cells

popt, _ = curve_fit(model_func, X_data, y_data)

y_fit = model_func(X_data, *popt)

\# FOURIER ANALYSIS OF TUMOUR DYNAMICS

dt = time[1] - time[0]

steps = len(time)

yf = fft(tumour_cells)

xf = fftfreq(steps, dt)[:steps//2]

amplitude = 2.0 / steps * np.abs(yf[:steps//2])

\# PLOT FIGURE 1: Tumour and NSCs Dynamics

plt.figure(figsize=(10, 6))

plt.plot(time, tumour_cells, label='Tumour Cells', color='crimson', linewidth=2)

plt.plot(time, nscs, label='NSCs', color='navy', linewidth=2)

plt.xlabel('Time (days)')

plt.ylabel('Normalized density')

plt.title('Figure 1: Baseline tumour-NSC dynamics')

plt.legend()

plt.grid(True, linestyle='--', alpha=0.4)

plt.tight_layout()

plt.savefig('figure1_tumour_nsc_dynamics.png', dpi=300)

plt.close()

\# PLOT FIGURE 2: Descriptive regression fit

\# ---------------------------------------------

plt.figure(figsize=(10, 6))

plt.plot(time, tumour_cells, label='Simulated tumour trajectory')

plt.plot(time, y_fit, '--', label='Non-linear regression fit')

plt.xlabel('Time (days)')

plt.ylabel('Normalized tumour density')

plt.title('Figure 2: Descriptive non-linear regression fit')

plt.legend()

plt.grid(True, linestyle='--', alpha=0.4)

plt.tight_layout()

plt.savefig('figure2_regression_fit.png', dpi=300)

plt.close()

\# PLOT FIGURE 3: Fourier spectrum

plt.figure(figsize=(10, 5))

plt.plot(xf, amplitude)

plt.xlabel('Frequency (day^-1)')

plt.ylabel('Amplitude')

plt.title('Figure 3: Fourier spectrum of tumour dynamics')

plt.grid(True, linestyle='--', alpha=0.3)

plt.tight_layout()

plt.savefig('figure3_fourier_spectrum.png', dpi=300)

plt.close()

\# FIGURES 4-6: sensitivity analyses (same baseline model)

r, alpha0, beta0, gamma = 0.20, 0.05, 0.35, 0.30

T0, N0 = 0.50, 0.40

def simulate_sensitivity(alpha=alpha0, beta=beta0):

T = np.zeros_like(time); N = np.zeros_like(time)

T[0], N[0] = T0, N0

for k in range(len(time)-1):

T[k+1] = T[k] + dt*(r*T[k]*(1-T[k]) - beta*T[k]*N[k])

N[k+1] = N[k] + dt*(alpha*N[k]*(1-N[k]) - gamma*T[k]*N[k])

return T, N

for a in [0.02, 0.05, 0.08, 0.10]: plt.plot(time, simulate_sensitivity(alpha=a)[0], label=f'alpha={a:.2f}')

plt.xlabel('Time (days)'); plt.ylabel('Normalized tumour density'); plt.legend(); plt.tight_layout(); plt.savefig('figure4_alpha_sensitivity.png', dpi=300); plt.close()

for b in [0.08, 0.20, 0.35, 0.50]: plt.plot(time, simulate_sensitivity(beta=b)[0], label=f'beta={b:.2f}')

plt.xlabel('Time (days)'); plt.ylabel('Normalized tumour density'); plt.legend(); plt.tight_layout(); plt.savefig('figure5_beta_sensitivity.png', dpi=300); plt.close()

alphas = np.linspace(0.02, 0.10, 41); betas = np.linspace(0.08, 0.50, 43)

Z = np.array([[simulate_sensitivity(alpha=a, beta=b)[0][-1] for b in betas] for a in alphas])

B, A = np.meshgrid(betas, alphas); plt.contourf(B, A, Z, levels=18); plt.colorbar(label="T20")

plt.xlabel('beta (day^-1)'); plt.ylabel('alpha (day^-1)'); plt.tight_layout(); plt.savefig('figure6_joint_sensitivity.png', dpi=300); plt.close()
